# CD276 as a promising diagnostic and prognostic biomarker for bladder cancer through bioinformatics and clinical research

**DOI:** 10.3389/fonc.2024.1445526

**Published:** 2024-09-10

**Authors:** Qi Zhou, Jianhao Xu, Xuelei Chen, Jun Ouyang, Caiping Mao, Zhiyu Zhang

**Affiliations:** ^1^ Department of Reproductive Medicine Center, The First Affiliated Hospital of Soochow University, Suzhou, China; ^2^ Department of Pathology, The First People’s Hospital of Kunshan, Suzhou, China; ^3^ Department of Urology, The First Affiliated Hospital of Soochow University, Suzhou, China

**Keywords:** CD276 expression, bladder cancer, prognosis, immunohistochemistry, bioinformatics database

## Abstract

**Objective:**

To assess CD276 expression and explore its relationship with the clinicopathological characteristics and prognosis of patients with bladder cancer.

**Methods:**

In total, RNA-sequencing data and clinical profiles of 436 bladder cancer cases from The Cancer Genome Atlas (TCGA) were assessed using the University of California Santa Cruz Xena (UCSC) platform. We compared the CD276 levels in cancerous and adjacent normal tissues and used the R software for statistical association with the clinical stage, grade, and survival (the overall survival, disease-specific survival, and progression-free survival). A single-gene GSEA analysis on TCGA-BLCA data was performed to explore potential pathways through which CD276 might influence bladder cancer. Additionally, CD276 expression was analyzed by comparing data from 9 cancerous tissues and 3 adjacent normal tissues in the GEO dataset GSE7476. Furthermore, we analyzed 133 cancerous bladder and adjacent tissue samples from the Soochow University Hospital, collected between January 1, 2016, and September 30, 2022, to assess the CD276 protein expression using immunohistochemistry. We examined the relationship between tumor CD276 levels and clinical outcomes and prognosis of bladder cancer.

**Results:**

Bioinformatic analysis revealed elevated CD276 expression in tumors compared to that in adjacent tissues (p<0.05), correlating with poor survival. GSEA revealed that CD276 was significantly involved in extracellular matrix-related pathways. Immunohistochemistry confirmed CD276 overexpression in tumor tissues, with higher levels linked to advanced pathological grades and worse prognosis.

**Conclusion:**

CD276 is markedly upregulated in bladder cancer and associated with severe pathological features, advanced disease, potential for metastasis, and diminished survival rates. It may promote bladder cancer development and progression by influencing extracellular matrix-related-related pathways, making it a viable diagnostic and prognostic biomarker for bladder cancer.

## 1 Introduction

Bladder cancer (BLCA) is among the most prevalent malignancies of the urinary tract worldwide, with approximately 386,000 new cases and 150,000 deaths per year (1–3). Incidence of urinary system cancers has increased, with BLCA being the fifth leading cause of cancer-related deaths in China. Urothelial carcinoma constitutes more than 95% of all BLCA cases, of which non-muscle invasive BLCA (NMIBC) represents 75-80% of all BLCA cases, known for its tendency to recur and progress, especially when limited to the mucosa (Ta) or submucosa (T1) (4, 5). Remarkably, in recent decades, China has experienced a swift increase in both the incidence and mortality rates of BLCA (6). Despite advances in treatment, the outlook for patients remains grim (7). Moreover, BLCA is more prevalent in urban areas than in rural settings (8).

Recent advances have revealed potential targets for the early detection, treatment, and prognostic evaluation of cancer. One such target in the B7 superfamily is the B7-Homologue 3 molecule (B7-H3, also known as the cluster of differentiation 276 [CD276]). This immunoglobulin family protein, discovered in 2001 by Andrei et al., plays an important role in modulating T-cell-driven immune responses and is a member of the B7/CD28 clan of the immune costimulatory molecules. CD276 shares 20-27% amino acid similarity with its B7 kin and exists in two isoforms: 2IgB7-H3 and 4IgB7-H3 (9, 10). Research has established that CD276 can markedly dampen T cell activity, positioning it as a prototypical immune checkpoint molecule (11, 12). Various tumor cells, vascular endothelial cells within tumors, and immune infiltrates such as dendritic cells and macrophages exhibit widespread and aberrant expression of the inhibitory molecule CD276. In contrast, its expression in the normal tissues is reported to be negligible (13, 14). Programmed cell death ligand 1 (PD-L1, also known as B7-H1) was initially identified in antigen-presenting cells, where its binding to the T-cell PD-1 receptor transmits a signal that curbs the immune response. Subsequently, this inhibitory mechanism was also observed in malignant cells; by expressing PD-L1, they interacted with T cell PD-1 to impair the immune response to tumors, enabling immune escape (15). Similarly, CD276 exerts a PD-L1-like suppressive effect in T cells (16).

Despite the undiscovered and unconfirmed receptors for human CD276, its precise immune and biological roles and mechanisms of action remain largely unknown. Nonetheless, research indicates that CD276, functioning as a “membrane molecule,” might facilitate “reverse signaling” by relaying signals to the cells in which it is expressed (17, 18).

CD276 generally serves as an activator of T cells. However, it can also act as a T cell inhibitor, potentially facilitating cancer cell invasiveness and proliferation (13–18). Additionally, it plays a role in cell-to-cell adhesion and interacts with the extracellular matrix (ECM) (19). In this study, we thoroughly examined CD276 expression to investigate its association with the prognosis and immune infiltration in BLCA, providing novel insights into potential diagnostic and therapeutic strategies for bladder urothelial carcinoma.

## 2 Materials and methods

### 2.1 Bioinformatics data description

#### 2.1.1 TCGA sample data collection

Using the University of California Santa Cruz (UCSC) Xena platform (https://xena.ucsc.edu/), we acquired RNA sequencing expression profiles and pertinent clinical data for BLCA from The Cancer Genome Atlas (TCGA) database. We determined CD276 levels in bladder tumors and assessed their associations with patient survival outcomes, specifically considering the overall survival (OS), disease-specific survival (DSS), and progression-free survival (PFS). The dataset included clinical information on 408 individuals with BLCA with 19 adjacent non-tumor tissue samples and 436 patients with BLCA.

#### 2.1.2 Association analysis between CD276 and clinic-pathological features of bladder cancer

The CD276 expression profiles in bladder tumor samples were integrated with the corresponding clinical data using the R language and the ‘ggpubr’ package (**Table 1**). This analysis investigated whether CD276 expression levels varied according to patient characteristics, such as sex, age, pathological grade, and tumor stage. For the sex-based analysis, the cohort was classified into 317 male and 119 female patients. Age was categorized using a median cutoff of 69 years, creating an older group comprising 209 patients and a younger group comprising 227 patients. With respect to the postoperative pathological grade, patients were grouped into high-grade urothelial carcinoma (413 cases) and low-grade urothelial carcinoma (23 cases). Tumor staging considered the depth of tumor invasion, occurrence of lymph node metastasis, and presence of distant metastasis, leading to a segregation of patients into either early stage (stage I+II, 140 cases with neither lymph node nor distant metastasis) or advanced stage (stage III+IV, 296 cases with lymph node metastasis or distant metastasis). This stratification facilitated targeted exploration of CD276 expression across different stages of tumor progression.

**Table 1:** Clinical information of bladder cancer patients in TCGA database.

Factors	Cases	Percentage (%)
Sex		
Female	119	27.29
Male	317	72.71
Age (years)		
>69	209	47.94
<=69	227	52.06
Grade		
High	413	94.72
Low	21	4.82
Unknow	2	0.46
Stage		
I+II	140	32.11
III+IV	295	67.66
Unknow	1	0.23

TCGA, The Cancer Genome Atlas.

#### 2.1.3 Expression of CD276 in bladder cancer and its prognostic analysis

The patients were stratified into high- and low-expression groups based on whether their CD276 expression levels were above or below the median. We applied the R language and employed the ‘survival, ‘surveyor,’ ‘survminer’ and ‘lima’ packages to depict the survival outcomes of these patients. This enabled the analysis of the association between CD276 expression and the key survival indicators OS, DSS, and PFS.

#### 2.1.4 GEO sample data collection

The dataset GSE7476 was obtained from the Gene Expression Omnibus (GEO) database (http://www.ncbi.nlm.nih.gov/geo/) by applying specific inclusion and exclusion criteria: (1) only studies on human tissues were included; (2) only BLCA studies were considered; (3) studies without a control group were excluded; and (4) only mRNA-level arrays were included, while dual-channel microarray studies were excluded. Differentially expressed genes (DEGs) between BLCA and normal samples were identified and analyzed using R language software. DEGs were defined as samples with corrected p-values <0.05 and log fold changes (FC) >1. The GSE7476 dataset contained 9 bladder cancer tissues and 3 adjacent normal tissues. Differences between the Tumor Group and the Normal Group were assessed using the Wilcoxon rank-sum test.

#### 2.1.5 Single-cell gene set enrichment analysis of CD276

Using data from TCGA-BLCA, we screened for differentially expressed genes between ccRCC and control samples with the DESeq2 package in R. Pearson association analysis identified 2,951 genes with an absolute association coefficient of CD276 >1 and a P-value <0.05. These genes were considered associated genes. They underwent enrichment analysis, with the top 10 related pathways visualized. We then employed the gene set enrichment analysis (GSEA) package in R to identify and visualize highly associated pathways.

### 2.2 Clinical data description

#### 2.2.1 Clinical sample collection

This study was a retrospective analysis involving 133 BLCA patients from the First Affiliated Hospital of Soochow University, evaluated between January 1, 2016, to September 30, 2022, with follow-up extending to close to 2023. This study was approved by the Ethics Committee of the First Affiliated Hospital of Soochow University (approval no. 283, 2024). Written informed consent was obtained from all participants before they enrolled in the study. This study adhered to the principles of the 2013 Declaration of Helsinki.

#### 2.2.2 Inclusion criteria

The inclusion criteria for the enrolled patients were as follows: (1) pathologically confirmed diagnosis of urothelial carcinoma; (2) complete clinical and follow-up data with willingness to participate in the study; (3) no concurrent malignant tumors or immunological diseases, including acquired immune deficiency syndrome; and (4) absence of infectious diseases prior to surgery and no prior exposure to radiation, chemotherapy, or other adjuvant treatments.

#### 2.2.3 Immunohistochemistry

Tissue samples were collected from 133 patients with BLCA, along with healthy tissues from neighboring areas, ensuring a minimum two-centimeter distance from the tumor border and confirming the absence of cancer cells post-surgery. These samples were used for IHC staining as described in our previous study (20). Briefly, IHC involves fixing tissue sections and treating them to expose antigens. Sections were then incubated with primary antibodies specific to the target proteins, followed by incubation with secondary antibodies conjugated with a detectable marker. After washing the sections, the presence of the target antigen was visualized using a colorimetric reaction or fluorescence and the tissue was counterstained for contrast before examination under a microscope. We utilized Aperio ImageScope software for the quantitative analysis of IHC staining.

CD276 was identified as yellow to brown granular staining of the cytoplasm at 100x magnification. To gauge positivity, ten random fields at 200x magnification were inspected, using a scoring system that combined the proportion of stained cells and staining intensity. Positivity percentages were scored as follows: less than 5% scored zero, 5–30% scored one, 31–60% two, and > 60% scored, three. Similarly, the intensity was scored from zero (no color) to three (dark brown). Multiplying these scores provided the final assessment; scores below two indicated negative results, while two or more were positive (21).

#### 2.2.4 Relationship between CD276 expression and survival of BLCA patients

The postoperative survival data for a cohort of 133 patients with BLCA were meticulously examined, with the follow-up extended until 2023. In this study, we investigated the association between CD276 expression and patient outcomes, specifically PFS and OS.

### 2.3 Statistical analysis

The gene expression data were normalized using log transformations. Data visualization was performed using the R software (version 4.2.1) along with auxiliary packages incorporating tools such as stats, limma, ggpubr, survival, survey, survminer, surveillance, ggplot2, DESeq2, and edgeR. Normal distributions are delineated by mean ± standard deviation (SD) and analyzed using t-tests. Alternatively, non-normally distributed data were expressed as medians (interquartile range, IQR) and examined using the Wilcoxon rank-sum test. The chi-squared test was used for categorical data assessment. Kaplan-Meier analysis facilitated the creation of survival curves, while the log-rank test was used for survival data comparison. Statistical significance was set at p < 0.05.

## 3 Results

### 3.1 The expression of CD276 is elevated in bladder cancer tissues in TCGA database

**Figure 1:**
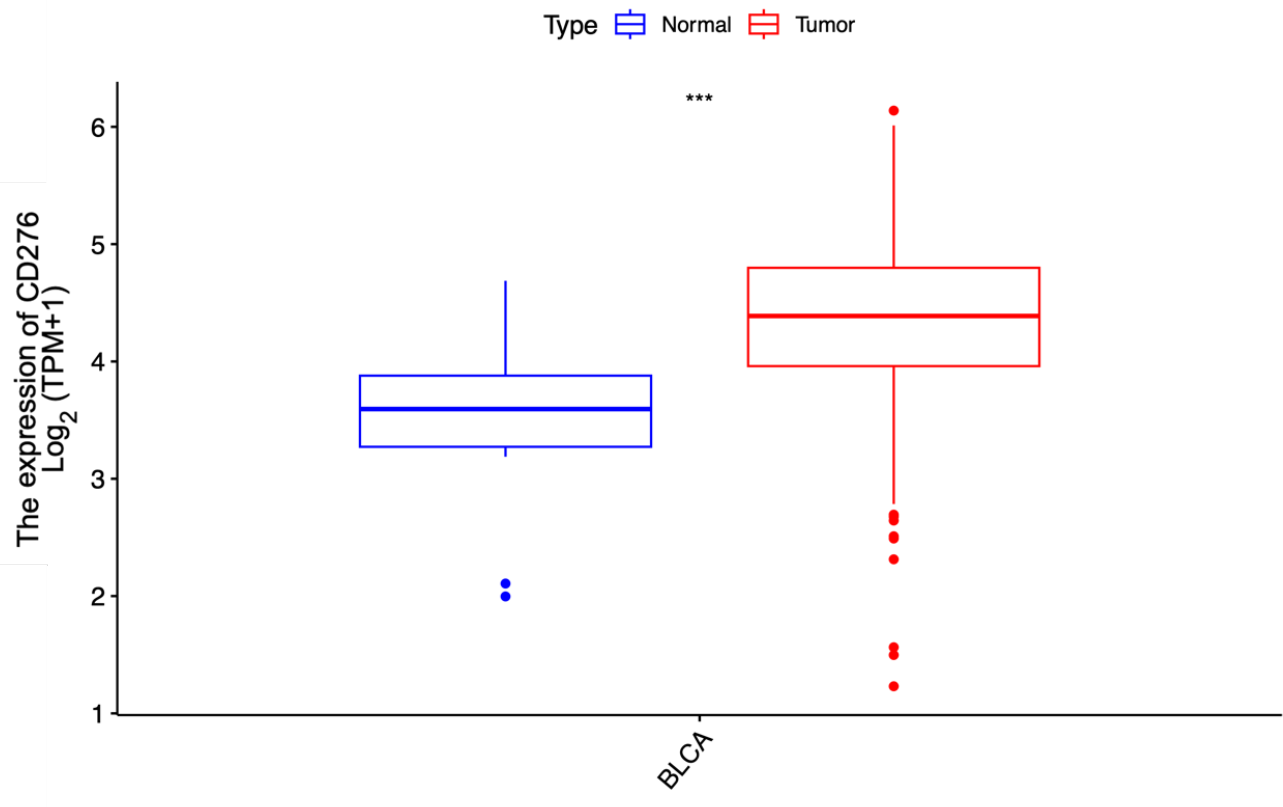
Expression of CD276 in bladder cancer tissues and para-cancer tissues in the TCGA database and the difference between the two groups. A significant difference was noted in the expression level of CD276 between 408 tumor tissues and 19 para-cancer tissues (p<0.001). CD276, cluster of differentiation 276; TCGA, The Cancer Genome Atlas, TPM, Transcripts Per Million; ***, p<0.001.

In this study, we assessed 408 BLCA specimens and 19 noncancerous tissue samples from the TCGA-BLCA dataset. As depicted in **Figure 1**, the results demonstrated that the median CD276 expression level in cancerous tissues was markedly elevated at 4.703, whereas in normal bladder tissues, the median expression was notably lower at 3.594. This pronounced discrepancy clearly indicates that CD276 is overexpressed in BLCA tissues compared with adjacent non-tumor tissues. Statistical analysis confirmed a significant difference in the CD276 expression between malignant bladder tissues and adjacent healthy tissues (p<0.001, **Figure 1**).

### 3.2 Association analysis between CD276 and gender, age, pathological grading, and staging of bladder tumors (436 cases) in TCGA database

**Figure 2:**
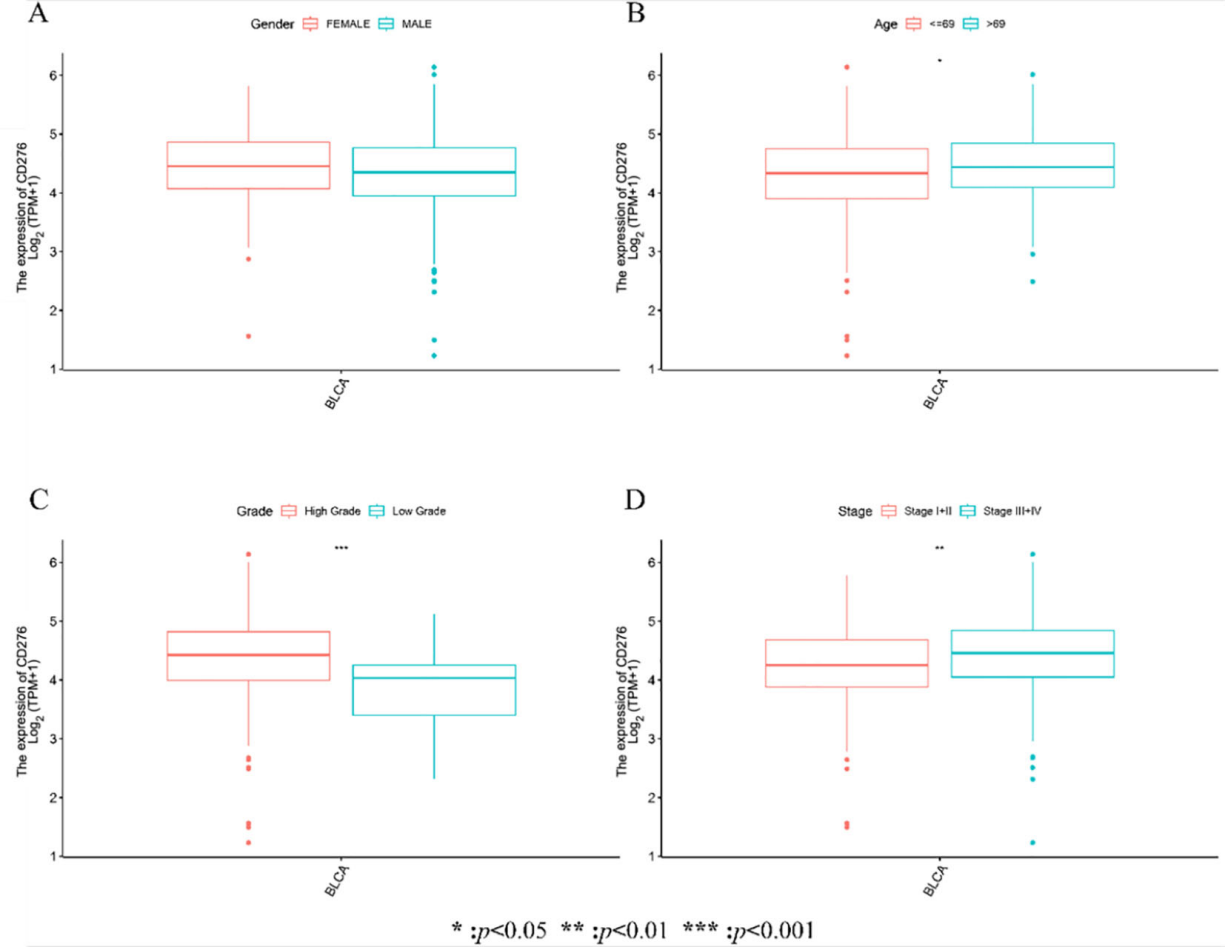
Expression and relationship of CD276 with different clinical characteristics in bladder cancer in TCGA database. **(A)**CD276 Expression levels in bladder tumor patients of different genders. **(B)** Expression level of CD276 in bladder tumors of different age groups (*p*<0.05). **(C)** Expression level of CD276 in bladder tumor tissues with different pathological grades (*p*<0.001). **(D)** Expression level of CD276 in bladder tumor tissues of different staging groups (*p*<0.01). CD276, cluster of differentiation 276; TCGA, The Cancer Genome Atlas; BLCA, bladder cancer, TPM, Transcripts Per Million.

Integrating CD276 expression data with clinical characteristics of BLCA patients from the (TCGA) database, we examined the variability of CD276 expression across various demographic and pathological variables. Our analysis of sex-specific expression revealed that CD276 levels remained remarkably consistent across sexes, showing no statistical significance in this disparity (**Figure 2A**). In contrast, further investigation into age, pathological grades, and tumor stages highlighted a clear pattern of differential CD276 expression; CD276 levels were notably higher in older patients, suggesting a potential age-related increase in expression (**Figure 2B**, p<0.05). Examining the pathological grades, we discovered a pronounced elevation of CD276 in high-grade urothelial carcinoma compared to its lower expression in its low-grade counterparts, with this variation being statistically significant (**Figure 2C**, p<0.001). Additionally, staging analysis revealed that CD276 expression intensified in the middle-to-late stages, especially in cases with lymph node involvement or distant metastasis. In contrast, early stage and non-metastatic tumors manifested lower CD276 levels, with the difference between advanced and early-stage patients being significant (**Figure 2D**, p<0.01). These findings indicate that high CD276 expression is significantly associated with advanced age, the presence of distant metastasis, and advanced disease stages, signifying deeper muscle invasion and a less favorable prognosis in patients with BLCA.

### 3.3 Survival analysis of BLCA patients between high expression and low expression of CD276 in TCGA database

**Figure 3:**
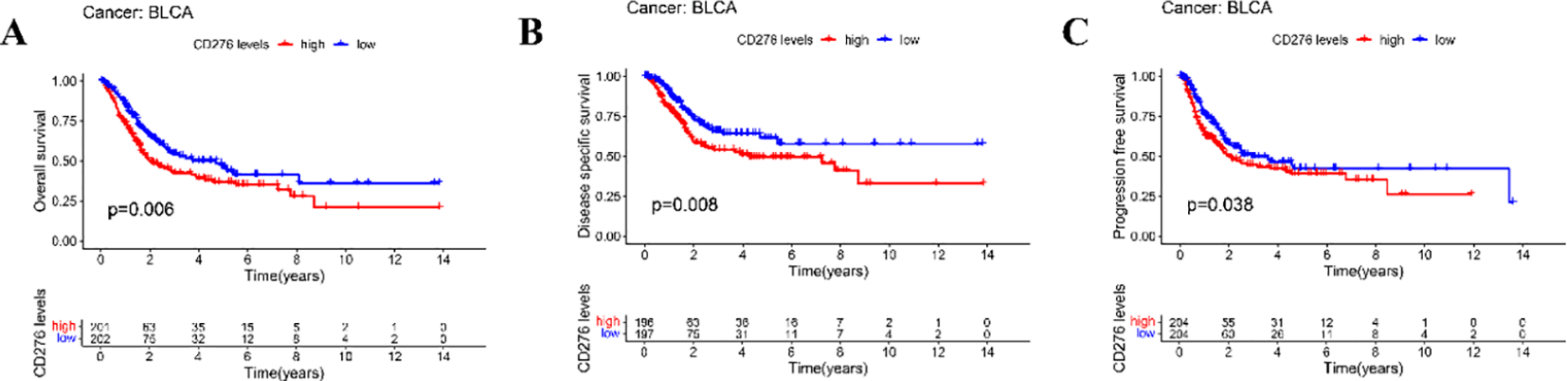
Relationship between the expression of CD276 in bladder cancer and survival of patients in TCGA database. **(A)** Association between the expression of CD276 and OS in bladder cancer patients(*p*=0.006). **(B)** Association between CD276 expression and DSS in bladder cancer patients (*p*=0.008). **(C)** Association between the expression of CD276 and PFS in bladder cancer patients(*p*=0.038). CD276, cluster of differentiation 276; TCGA, The Cancer Genome Atlas; OS, overall survival; DSS, disease-specific survival; PFS, progression-free survival; BLCA, bladder cancer.

Our survival analyses indicated that elevated CD276 expression associated with the OS, DSS, and PFS among patients with BLCA (**Figure 3**). Specifically, we observed that increased levels of CD276 portend a diminished OS, with the category expressing higher CD276 levels, revealing significantly lower survival rates compared to the low-expression cohort (**Figure 3A**, p=0.006). A similar trend was noted for both DSS and PFS, where the high CD276 expression group exhibited markedly lower survival rates for DSS (**Figure 3B**, p=0.008) and PFS (**Figure 3C**, p=0.038). Based on these findings, we inferred a strong association between high CD276 expression and reduced OS and suboptimal prognostic outcomes in patients with BLCA.

### 3.4 The expression of CD276 is elevated in bladder cancer tissues in GEO database

**Figure 4:**
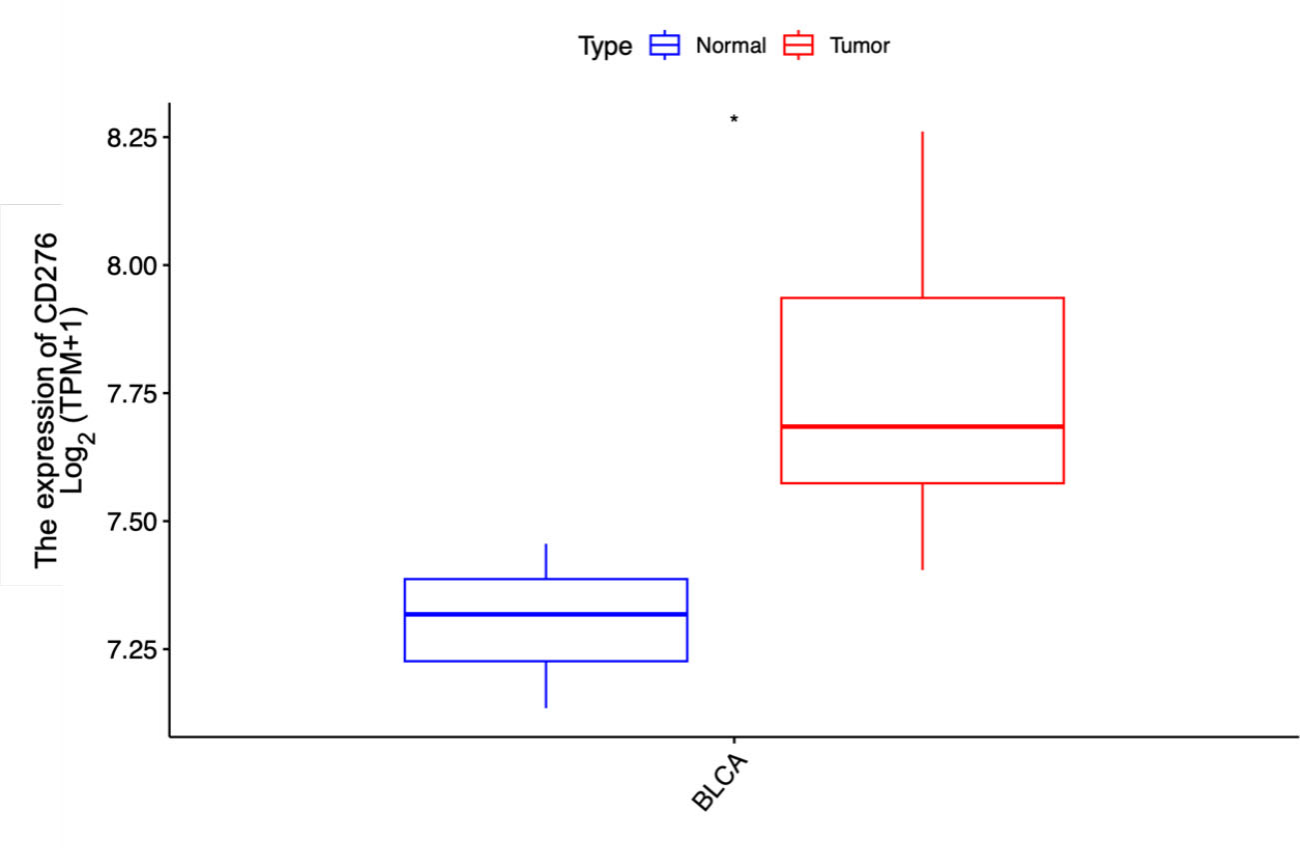
Expression of CD276 in bladder cancer tissues and para-cancer tissues in the GEO database and the difference between the two groups. The expression level of CD276 in 9 bladder tumor tissues was higher than that in 3 para-cancer tissues. CD276, cluster of differentiation 276; GEO, Gene Expression Omnibus, TPM, Transcripts Per Million; *, p<0.05.

We also extracted CD276 expression data for 9 bladder cancer tissues and 3 adjacent normal tissues from the GEO dataset GSE7476. Our analysis further confirmed that CD276 expression was significantly higher in bladder cancer tissues compared to adjacent normal tissues (7.769 ± 0.276 vs. 7.303 ± 0.161, p=0.022, **Figure 4**).

### 3.5 Gene set enrichment analysis for pathways related to CD276 in patients with bladder cancer

**Figure 5:**
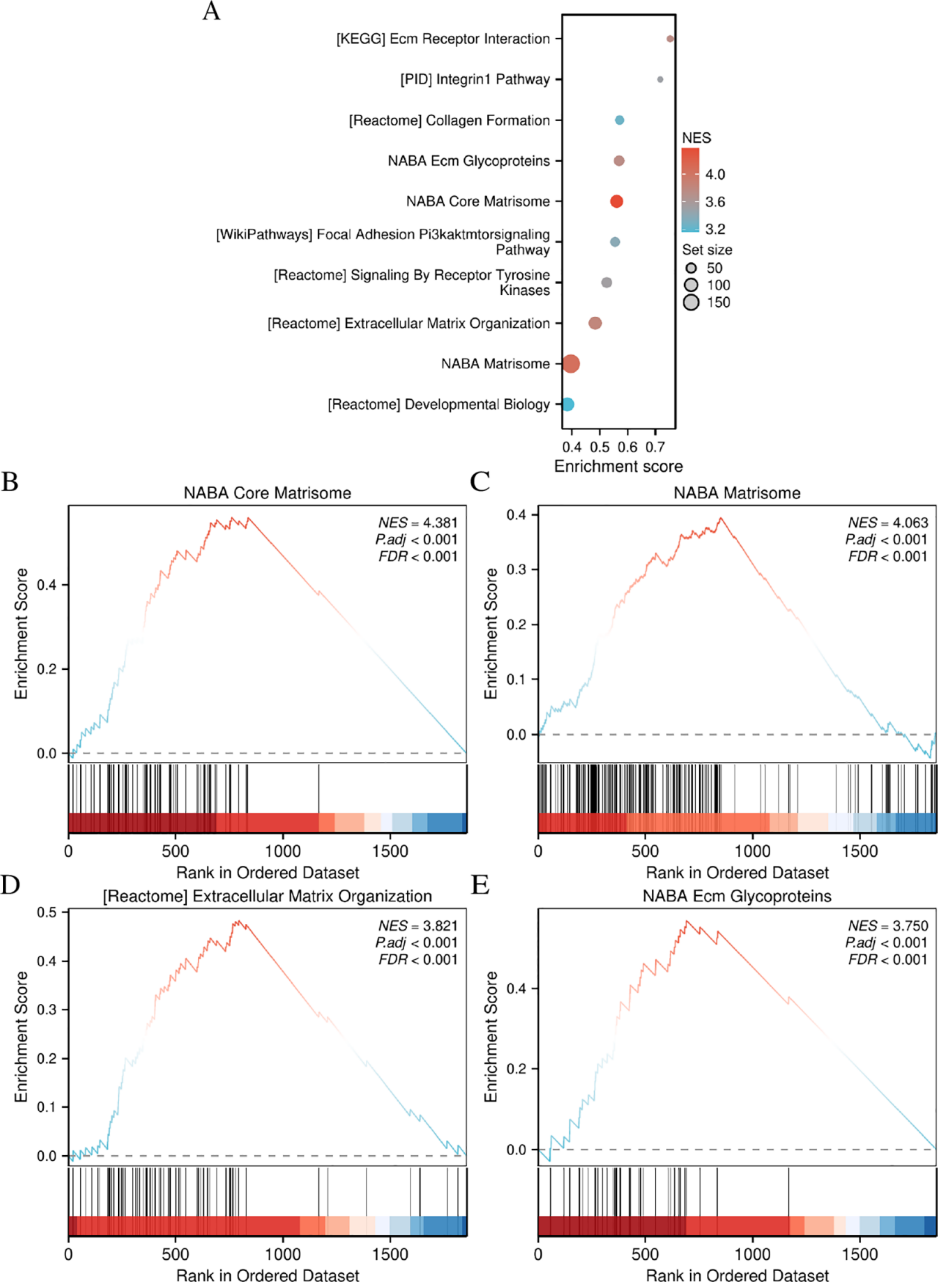
The high CD276 expression group in patients with bladder cancer based on GSEA. **(A)** The top 10 pathways enriched in the high CD276 expression data set; **(B–E)** The high CD276 expression data set was enriched in core matrisome, matrisome, extracellular matrix organization, and ECM glycoproteins pathways. CD276, cluster of differentiation 276; GESA, gene set enrichment analysis.

To elucidate the regulatory mechanism of CD276 in bladder cancer, GSEA and pathway visualization were conducted based on the differentially expressed genes between BLCA tissues and control samples (**Figure 5A**). GSEA analysis revealed that high CD276 expression was significantly enriched in core matrisome, matrisome, extracellular matrix organization, and ECM glycoproteins pathways (**Figures 5B–E**).

### 3.6 The relationship between the expression of CD276 in bladder cancer and clinic-pathological characteristics in clinical data

Upon dissecting the interplay between CD276 expression levels and a range of clinical attributes in 133 BLCA specimens, we found no major disparities in CD276 expression based on sex, age, body mass index (BMI), history of hypertension (HBP), diabetes mellitus (DM), hyperlipidemia, tumor diameter, or the presence of multiple tumors (**Table 2**, p>0.05). However, a significant divergence was observed when the CD276 expression was compared across different pathological grades. Specifically, high-grade urothelial carcinoma showed elevated CD276 expression, reinforcing the connection between high CD276 levels and more aggressive cancer grades (**Table 2**, p<0.05). By delving deeper into the relationship between CD276 expression and postoperative tumor staging, we discovered an increasing trend in CD276 levels in relation to advancing tumor stages. Statistical analyses confirmed that these differences in expression were discernible across different stages, indicating a broader implication of CD276 in the progression of BLCA (**Table 2**, p<0.05).

**Table 2:** Association between expression of CD276 and clinical features of bladder urothelial cell carcinoma.

Characteristics	Positive	Negative	t/χ^2^	p value
n	104	29		
Sex, n (%)			0.60	0.439
Male	79 (75.96%)	24 (82.76%)		
Female	25 (24.04%)	5 (17.24%)		
Age (year), mean ± sd	65.94 ± 12.17	69.00 ± 10.38	-1.23	0.220
BMI (kg/m2), mean ± sd	23.40 ± 3.04	22.44 ± 3.21	1.49	0.138
HBP, n (%)			0.28	0.595
Yes	56 (53.85%)	14 (48.28%)		
No	48 (46.15%)	15 (51.72%)		
DM, n (%)			0.09	0.768
Yes	19 (18.27%)	6 (20.69%)		
No	85 (81.73%)	23 (79.31%)		
Hyperlipemia, n (%)			0.09	0.763
Yes	28 (26.92%)	7 (24.14%)		
No	76 (73.08%)	22 (75.06%)		
Tumor Diameter(mm), median (IQR)	30 (25, 45)	30 (20, 35)	1.77	0.054
Tumor multiplicity, n (%)			3.85	0.050
Yes	80 (76.92%)	17 (58.62%)		
No	24 (23.08%)	12 (41.38%)		
Pathological Grading, n (%)			6.43	0.011
High Grade	79 (75.96%)	15 (51.72%)		
Low Grade	25 (24.04%)	14 (48.28%)		
T stage, n (%)			7.56	0.023
T2	71 (68.27%)	27 (93.10%)		
T3	21 (20.19%)	2 (6.90%)		
T4	12 (11.54%)	0 (0%)		

CD276, cluster of differentiation 276; BMI, body mass index; HBP, high blood pressure; DM, diabetes mellitus; SD, standard deviation; IQR, interquartile range.

### 3.7 The expression of CD276 in cancer and para-cancer tissues in clinical data

**Figure 6:**
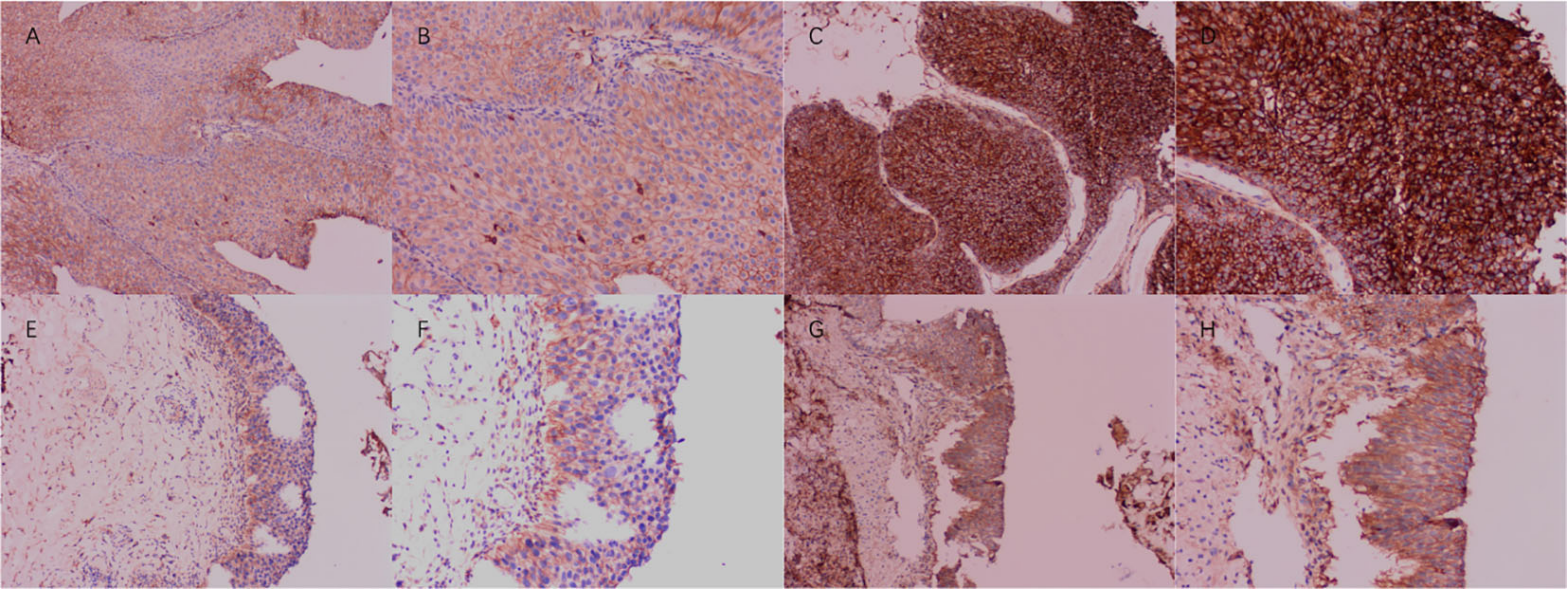
Expression of CD276 in bladder cancer and para-cancer tissues. **(A)** Negative expression of CD276 in bladder cancer (100x). **(B)** Negative expression of CD276 in bladder cancer (200x). **(C)** Positive expression of CD276 in bladder cancer (100x). **(D)** Positive expression of CD276 in bladder cancer (200x). **(E)** Negative expression of CD276 in para-cancer tissues (100x). **(F)** Negative expression of CD276 in para-cancer tissues (200x). **(G)** Positive expression of CD276 in para-cancer tissues (100x). **(H)** Positive expression of CD276 in para-cancer tissues (200x). CD276, cluster of differentiation 276.

IHC analysis was conducted on a cohort of 133 paired samples comprising BLCA tissues and their adjacent non-tumor counterparts, as depicted in **Figure 6**. IHC evaluation revealed that the prevalence of CD276 expression was substantially elevated in the cancerous cohort (78.20%) compared with that in the non-cancerous cohort (17.29%). These findings underscore a markedly increased expression of CD276 in cancerous tissues relative to neighboring non-cancerous tissues, with the difference being statistically significant (χ²=98.86, p<0.001, as shown in **Table 3**).

**Table 3:** CD276 expression in bladder urothelial cell carcinoma and para-cancer tissues.

Tissue	CD276 expression [case (%)]		χ^2^	p value
	Positive	Negative		
Tumor tissues	104(78.20)	29(21.80)	98.86	<0.001
Para-cancer tissues	23(17.29)	110(82.71)

CD276, cluster of differentiation 276.

### 3.8 Relationship between CD276 expression and survival of BLCA patients in clinical data

**Figure 7:**
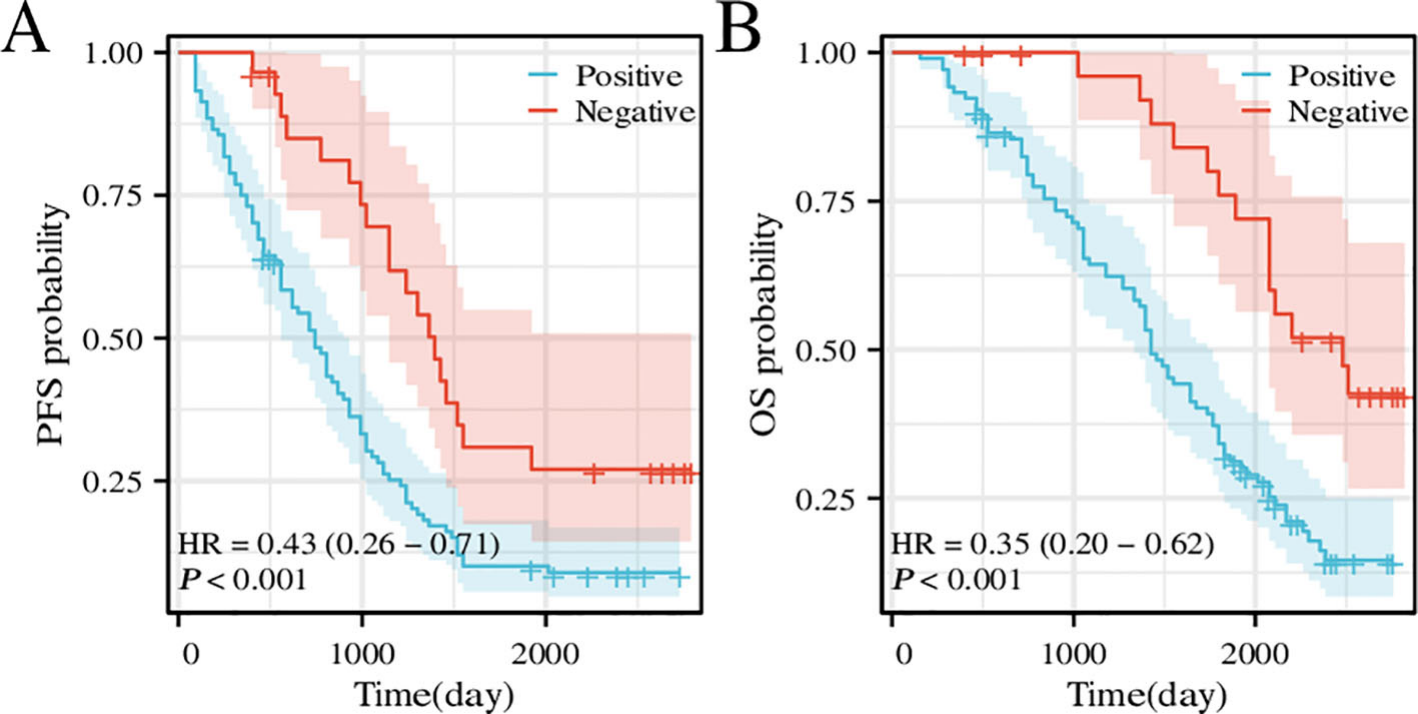
Survival curve of PFS and OS for patients with high or low CD276 expression in bladder cancer. **(A)** Survival curve of PFS for patients with high or low CD276 expression in bladder cancer. **(B)** Survival curve of OS for patients with high or low CD276 expression in bladder cancer. CD276, cluster of differentiation 276; PFS, progress-free survival; OS, overall survival; HR, hazard ratio.

Examination of postoperative follow-up data from 133 patients with BLCA revealed an association between CD276 expression and survival. An analysis focusing on the association between CD276 levels and PFS revealed that elevated CD276 expression was associated with diminished PFS (**Figure 7A**, p<0.001, hazard ratio [HR]=0.43). Similarly, when assessing the impact of CD276 expression on the OS, a pattern was observed in which increased CD276 levels corresponded to reduced OS (**Figure 7B**, p<0.001, HR=0.35). Elevated CD276 expression is associated with patient survival outcomes in BLCA, with higher levels indicating a truncated survival span and a poorer prognostic outcome.

## 4 Discussion

BLCA is the most common urinary tract malignancies in China, and postoperative chemotherapy is the primary strategy to combat this cancer. However, resistance to chemotherapy often results in treatment failure, posing a severe risk to patient survival. The introduction of immunosuppressive agents has significantly altered the treatment landscape for numerous solid cancers (22), although the effectiveness of such immunotherapies is restricted to selective tumor profiles (23). Among the various immune checkpoints that have been investigated, CD276 has attracted considerable attention. Blocking CD276 has shown promise for the treatment of various solid cancers as it is a protein prolific protein across tumor sites, affecting tumor cells, dendritic cells, and macrophages. Thus, its inhibition can have far-reaching effects. CD276 is an immunomodulatory protein that promotes cancer cell growth. It also inhibits immune cell-mediated tumor responses and promotes cancer cell differentiation (24). Regulatory T-cells (Tregs) induce interleukin-10 and CD276 expression in dendritic cells (DCs), conferring immunosuppressive properties. Upon Treg exposure, DCs upregulate CD276 and reduce major histocompatibility complex peptide complex presentation, thereby weakening T cell activation. Hence, CD276 is critical for the T cell-mediated suppression of DCs. However, the mechanism of action of CD276 is yet to be fully elucidated. Moreover, CD276 is implicated in pathways such as PI3K/AKT, NF-KB, Ras/Raf/MEK/MAPK, JAK2/STAT3, and glucose metabolism (24).

In recent years, substantial advancements have been made in the field of tumor immunotherapy, notably the triumphant integration of the B7 family molecule PD-L1 and its receptor PD-1 inhibitors into clinical practice, heralding new perspectives for cancer treatment. B7-H3 is a newly identified member of the B7 lineage and is frequently upregulated in a wide spectrum of human malignancies, where its elevated expression often parallels the progression, prognosis, or classification of tumors (25). Although the precise biological role and mechanisms underlying its contribution to immune evasion are the subjects of ongoing investigation, CD276’s pronounced expression in cancerous tissues and vasculature, including its prevalence in as many as 98% of renal cell carcinoma (RCC) blood vessels (26), underscores its potential as a distinctive and potent vessel-specific marker of cancer. Owing to its close association with the biological attributes of tumors, CD276 shows promise as an emergent biomarker target for cancer diagnostics, increasingly capturing the attention of the medical community.

This study investigated the association between CD276 expression within BLCA and patient prognosis, validating CD276 expression in these tumors through IHC assessment. Our research utilized RNA sequencing expression profiles and the corresponding clinical data for BLCA retrieved from TCGA database via the Xena platform. We scrutinized both the expression data and related clinical details. An initial review of CD276 expression in 408 BLCA specimens and 19 adjacent non-cancerous tissues revealed a markedly elevated level of CD276 in cancerous tissues (**Figure 1**, p<0.001). Data from another GEO dataset, GSE7476, further confirmed that CD276 expression is higher in bladder cancer tissues compared to adjacent normal tissues (**Figure 4**, p<0.05). Our subsequent analyses probed the relationship between CD276 expression in various samples and clinically relevant characteristics. We observed a uniform expression pattern of CD276 among patients with BLCA, irrespective of the sex. Nevertheless, enhanced levels of CD276 were more evident in older patients, those with high-grade urothelial carcinoma, and individuals exhibiting lymph node or distant metastases during advanced stages of the disease (**Figure 2**, p<0.05). This finding also supports the idea that elevated CD276 expression is indicative of advanced muscle invasion, distant metastasis, and unfavorable prognosis in individuals with BLCA. The association between high CD276 expression and OS was further corroborated by survival analysis, which identified a link between reduced survival odds in BLCA patients (**Figure 3**, p<0.05). We conducted a single-cell GSEA for CD276 to investigate its potential association with extracellular matrix-related pathways, including core matrisome, matrisome, extracellular matrix organization, and ECM glycoproteins pathways. The ECM is a component of all mammalian tissues, consisting predominantly of fibrous proteins like collagen, elastin and associated microfibrils, fibronectin, and laminins, embedded in a viscoelastic gel of anionic proteoglycan polymers. Beyond its structural role, the ECM performs various functions by influencing cell behaviors such as proliferation, adhesion, migration, and regulating cell differentiation and death as a crucial component of the cellular microenvironment (19, 27, 28). Bin Lim S et al. (27) reported an association between CD276 and the matrisome pathway in breast cancer. Additionally, Mei J et al. (28) found that CD276 expression had consistent associations with collagen signatures in bladder cancer, contributing to tumor metastasis and poor prognosis.

To reinforce our findings, we examined the association between CD276 expression levels and diverse clinical parameters in 133 BLCA samples obtained from the First Affiliated Hospital of Soochow University. Our analysis highlighted a more pronounced expression of CD276 in high-grade urothelial carcinoma, thereby upholding the established link between elevated CD276 levels and this aggressive cancer variant. Additionally, we observed a direct association between tumor stage advancement and increased CD276 expression (**Table 2**; p<0.05). Immunohistochemical assays revealed that CD276 was present in 78.20% of tumor specimens, as opposed to 17.29% of normal tissue samples. The CD276 expression is revealed to be significantly higher in tumor tissues compared to that in adjacent non-cancerous tissues (χ2 = 98.86, p<0.001, **Table 3**). This empirical evidence lends substantial weight to the initial conclusions.

In addition to CD276, which we have introduced, there are other urinary biomarkers for bladder cancer that have been used in clinical settings. For instance, the bladder tumor antigen (BTA) test has been utilized, but it shows a positive expression in only 54% of patients (29). Other reported gene expression measurements, such as CXCR2 and CDC2, are currently still in the experimental stages, including cell and animal studies (30, 31). These biomarkers have not yet been widely implemented in clinical practice, and their efficacy requires further validation through clinical trials. Beyond molecular biomarkers like CD276, recent studies have underscored the significance of the urinary microbiome in bladder cancer. Specifically, an analysis of patients undergoing transurethral resection of bladder tumors found an elevated presence of Porphyromonas and Porphyromonas somerae in morning urine samples from male bladder cancer patients, indicating their potential as specific predictive biomarkers (32). These findings support the hypothesis that a multifactorial diagnostic approach, combining molecular and microbiological biomarkers, could significantly improve the accuracy of bladder cancer diagnosis and prognosis.

Nevertheless, this study has certain limitations. Although our bioinformatics analysis was based on the TCGA and GEO databases, further validation of our findings through additional databases was still required. The clinical sample size was somewhat limited and originated from a single-center study. Consequently, the findings presented here should be treated with caution and further validated using larger-scale multicenter studies. Moving forward, we intend to delve deeper into the molecular mechanisms of action of CD276 in BLCA.

In conclusion, the findings of our study suggest that the CD276 expression is significantly associated with the prognostic outcomes of BLCA. Moreover, this expression associates with the disease pathological grade, stage, and occurrence of distant metastasis, demonstrating a robust predictive capacity for patient prognosis. Additionally, CD276 may promote the development and progression of bladder cancer through ECM-related pathways. These results further highlight CD276’s potential as an immunotherapeutic biomarker for BLCA.

## Data Availability

The datasets presented in this study can be found in online repositories. The names of the repository/repositories and accession number(s) can be found in the article/supplementary material.
